# Antidepressant-like Effect of *Oroxylum indicum* Seed Extract in Mice Model of Unpredictable Chronic Mild Stress

**DOI:** 10.3390/nu15224742

**Published:** 2023-11-10

**Authors:** Chorpeth Chalermwongkul, Charinya Khamphukdee, Juthamart Maneenet, Supawadee Daodee, Orawan Monthakantirat, Chantana Boonyarat, Yutthana Chotritthirong, Suresh Awale, Anake Kijjoa, Yaowared Chulikhit

**Affiliations:** 1 Graduated School of Pharmaceutical Sciences, Khon Kaen University, Khon Kaen 40002, Thailand; c.chorpeth@kkumail.com (C.C.); yutthana_ch@kkumail.com (Y.C.); 2 Division of Pharmacognosy and Toxicology, Faculty of Pharmaceutical Sciences, Khon Kaen University, Khon Kaen 40002, Thailand; charkh@kku.ac.th (C.K.); ankijjoa@icbas.up.pt (A.K.); 3 Division of Pharmaceutical Chemistry, Faculty of Pharmaceutical Sciences, Khon Kaen University, Khon Kaen 40002, Thailand; juthamart_pp@hotmail.com (J.M.); csupawad@kku.ac.th (S.D.); oramon@kku.ac.th (O.M.); chaboo@kku.ac.th (C.B.); 4 Natural Drug Discovery Laboratory, Institute of Natural Medicine, University of Toyama, 2630 Sugitani, Toyama 930-0154, Japan; suresh@inm.u-toyama.ac.jp; 5 ICBAS-Instituto de Ciências Biomédicas Abel Salazar and CIIMAR, Universidade do Porto, Rua de Jorge Viterbo Ferreira 228, 4050-313 Porto, Portugal

**Keywords:** baicalein, depression, HPA axis, *Oroxylum indicum*, unpredictable chronic mild stress

## Abstract

Major depressive disorder (MDD) is one life-threatening disorder that is prevalent worldwide. The evident etiology of this disease is still poorly understood. Currently, herbal medicine is gaining more interest as an alternative antidepressant. *Oroxylum indicum*, which is used in traditional medicine and contains a potential antidepressive compound, baicalein, could have an antidepressive property. An *in vitro* monoamine oxidase-A (MAO-A) inhibitory assay was used to preliminarily screening for the antidepressant effect of *O. indicum* seed (*OIS*) extract. Mice were subjected to unpredictable chronic mild stress (UCMS) for 6 weeks, and the daily administration of *OIS* extract started from week 4. The mechanisms involved in the antidepressive activity were investigated. The *OIS* extract significantly alleviated anhedonia and despair behaviors in the UCMS-induced mouse model via two possible pathways: (i) it normalized the HPA axis function via the restoration of negative feedback (decreased FKBP5 and increased GR expressions) and the reduction in the glucocorticoid-related negative gene (SGK-1), and (ii) it improved neurogenesis via the escalation of BDNF and CREB expressions in the hippocampus and the frontal cortex. In addition, an HPLC analysis of the *OIS* extract showed the presence of baicalin, baicalein, and chrysin as major constituents. All of the results obtained from this study emphasize the potential of *OIS* extract containing baicalin and baicalein as an effective and novel alternative treatment for MDD.

## 1. Introduction

According to the World Health Organization (WHO), more than 350 million people have already suffered from depression, 94% of whom manifest cognitive symptoms such as concentration difficulties, forgetfulness, and indecisiveness, which affect their aspects of life. Moreover, it is suggested that depression is associated with a higher likelihood of unnatural death due to suicide [1]. Even though the evident pathophysiology of depression remains largely uncharacterized, genetic factors and psychological stress events are clearly associated with major depressive disorder (MDD) [2]. The primary stress-related hormone in humans is cortisol (also corticosterone in rodents), which is regulated by the hypothalamic–pituitary–adrenal (HPA) axis. The excessive exposure to stressors leads to an alteration in HPA system regulation via the destruction of negative feedback, resulting in hypercortisolism found in patients with a mood disorder [3,4]. FK506 binding protein 51 (FKBP5), a co-chaperone in the glucocorticoid receptor (GR) complex, has a negative effect on GR signaling. When FKBP5 is bound to GR, it increases receptor resistance, which inhibits GR translocation to the nucleus, and negative feedback may not occur [5,6]. In addition, it is suggested that hypercortisolism secondarily downregulates GR expression, which could be ameliorated via antidepressants [7]. There is a lot of evidence demonstrating that HPA axis dysfunction could reduce neurotrophic proteins, such as brain-derived neurotrophic factor (BDNF), which contribute to neuronal cell death and the alteration of neurogenesis in the hippocampus [8]. Interestingly, the higher expression of serine/threonine-protein kinase 1 (SGK-1), another protein regulated by glucocorticoid (GC) activities, can weaken neuroplasticity in related brain regions [9].

**Figure 1:**
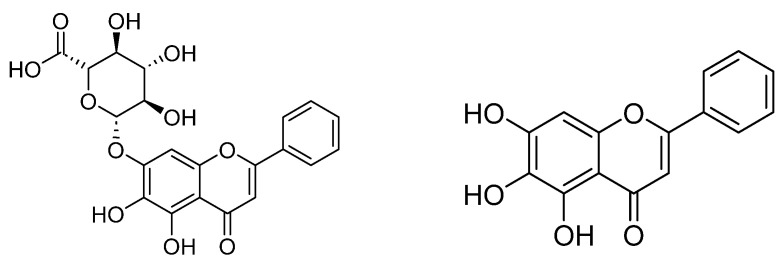
Structures of baicalin and its aglycone, baicalein.

Baicalin (baicalein-7-O-glucuronide) and its aglycone, baicalein (5,6,7-trihydroxyflavone), present the highest biological activities compared with other compounds, especially in the treatment of neurodegenerative diseases, such as depression or cognitive impairment (Figure 1) [10,11,12]. Various studies have suggested that baicalin ameliorates the depression-like behaviors in animal models with multiple mechanisms of action such as regulating the HPA axis, promoting neurogenesis, improving mitochondrial dysfunction, and suppressing neuronal inflammation and oxidative stress, as well as attenuating neuronal apoptosis [12]. These flavonoids are found in the root of the traditional Chinese medicine, *Scutellaria baicalensis* Georgi, one of the components of Xiao-chai-hu-tang (XCHT) that has been widely used clinically in depressive disorders in China [12]. In addition, XCHT has been shown to significantly ameliorate depressive-like behavior in a rat model by improving the serotoninergic function and neurotrophic factors in the hippocampus [12,13]. In Thailand, baicalin and baicalein can be found in *Oroxylum indicum* (L.) Kurz, a plant belonging to the family Bignoniaceae. It is an edible plant that has also been used in a traditional Thai medicine remedy for centuries. It is cultivated throughout Southeast and South Asian countries, and it is commonly known as “Trumpet tree” due to the shape of its flowers [14]. Every part of *O. indicum*, including the stem barks, pods, leaves, and seeds, exerts a myriad of pharmacological activities, such as antioxidant [15], anti-inflammatory [16,17], and neuroprotective [14] activities. The constituents that are mostly found in *O. indicum* are baicalein, baicalin (baicalein-7-O-glucoside), chrysin, and oroxylin-A [18]; however, in some cases, quercetin and kaempferol were also isolated [19]. *O. indicum*, which contains baicalein and other effective flavonoids, has been studied for its anticancer potential [20,21] and anti-inflammatory effect [22], but there was no scientific evidence reporting an antidepressive effect. Our preliminary studies exhibited that the *O. indicum* seed (*OIS*) extract contained the highest baicalin content when compared to the *O. indicum* pod and root extracts (Table S1 in Supplementary Material). For this reason, *O. indicum* seed (*OIS*) extract was investigated for its antidepressant effects on an UCMS-induced mouse model in the present study. Moreover, quality control was also performed to identify the active components in the *OIS* extract.

## 2. Materials and Methods

### 2.1. Preparation of the OIS Extract

Seeds of *O. indicum* were collected in Khon Kaen province, Thailand, and the plant material was identified by Dr. Prathan Leucha of the Faculty of Pharmaceutical Sciences, Khon Kaen University, Thailand. The herbarium voucher specimen was deposited at the herbarium of the Faculty of Pharmaceutical Sciences of Khon Kaen University, Thailand. Dried and powdered seeds of *O. indicum* (2 kg) were macerated in 95% ethanol (12 L) at room temperature for three days, and then filtered using filter paper to separate the solid residues from the ethanolic solution. The process was repeated three times, and the ethanolic solutions were pooled and evaporated using a rotary evaporator at 50 °C. The crude extract (340.2 g) was then freeze-dried and stored at −20 °C throughout the experiment.

### 2.2. Inhibitory Effect on Monoamine Oxidase-A (MAO-A) Enzyme

Monoamine oxidase enzymes (MAOs) are involved in the depletion of monoamine neurotransmitters such as serotonin (5-HT), dopamine (DA), and norepinephrine (NE). The antidepressant mechanism is involved in the inhibition of MAOs, especially the MAO-A isoform [23]. To preliminarily screen the antidepressant effect of the *OIS* extract, recombinant human MAO-A (Sigma-Aldrich, St. Louis, MO, USA) was used as the enzyme source, and kynuramine was used as a substrate to produce 4-hydroxyquinoline. The *OIS* extract (250 mg) was dissolved in DMSO (1000 µL) as a stock solution (250 mg/mL), and then a serial dilution was performed. Each sample (10 µL) was mixed with kynuramine (Sigma-Aldrich, St. Louis, MO, USA) (4.5 µL) and potassium phosphate buffer (234.75 µL). MAO-A enzyme (0.75 µL) was added to the mixture and kept at 37 °C for 20 min, after which 2N NaOH (200 µL) and water (500 µL) were added to stop the reaction. 4-Hydroxyquinoline was measured using the spectrofluorometric method at a wavelength of 310 nm for excitation and 400 nm for emission. The IC_50_ values were calculated by using GraphPad Prism ver. 8.4.3 software. Clorgyline (Sigma-Aldrich, St. Louis, MO, USA), a selective MAO-A inhibitor, was used as a positive control.

### 2.3. Animals

Male mice from the Institute of Cancer Research (ICR), bred by Nomura Siam International Company in Thailand, were used in the experiment. Five mice per cage were housed in transparent cages with free access to food and water under thermostatic conditions at 22 ± 2 °C, with constant humidity (45 ± 2%) and a 12 h light/dark cycle (light on 06:00 a.m. to 18:00 p.m.). The procedure was certified by the Animal Ethics Committee for Use and Care of Khon Kaen University (IACUC-KKU-104/64). All experiments were strictly implemented in accordance with the Guiding Principles for the Care and Use of Animals (NIH Publications No. 80–23, revised in 2011).

### 2.4. Unpredictable Chronic Mild Stress (UCMS) Paradigm

**Figure 2:**
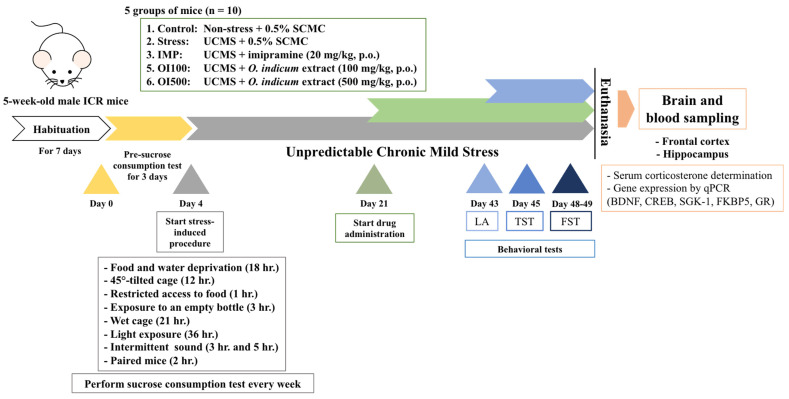
Schematic drawing of unpredictable chronic mild stress (UCMS) procedure.

As shown in Figure 2, mice were randomly divided into five groups; the first group was a non-stress group, which was housed in a normal environment, and the others were subjected to unpredictable mild stress according to the schedule over one week. The procedure was performed following the previous study [24]. Stressors consisted of 2 groups; two periods a week, such as 45° tilted cage (12 h), access to 5 small pieces of food (1 h), exposure to empty bottle (3 h), consistent light exposure (36 h), and subject to disturbed noise (3 and 5 h), while the other stressors were given once a week, such as wet cage (21 h), food and water deprivation (18 h), and paired mice from different cage (2 h). All of these stressors started at the first week and were repeated throughout the experiment.

### 2.5. Experimental Design and Drug Administration

After habituation, a sucrose preference test (SPT), a measurement of anhedonia behavior, was conducted with 2% *w/v* sucrose solution. All mice underwent adaptive training from day 1 to day 3, after 18 h of food and water deprivation, and each mouse was given 2% sucrose for 1 h. The amount of sucrose consumption during 1 h was recorded and calculated as the baseline of sucrose consumption in week 0. Mice were randomly divided into 5 groups (*n* = 10 each) as follows: (i) non-stress group, which was treated with 0.5% sodium carboxymethyl cellulose (SCMC, Mumbai, India) as vehicle (0.5% SCMC, 1 mL/kg, p.o.); (ii) UCMS-induced group, which was treated with vehicle (0.5% SCMC, 1 mL/kg, p.o.); (iii) the positive control group, which was the UCMS-induced group treated with imipramine hydrochloride (IMP) (Nacalai tesque, Inc., Kyoto, Japan) in 0.9% normal saline solution (NSS) (IMP, 20 mg/kg, p.o.); (iv) and (v) the experimental groups, which were the UCMS-induced groups treated with the *OIS* extract at 100 and 500 mg/kg p.o., respectively. Both selected doses were calculated based on the baicalin content in the *OIS* extract, which was analyzed using the HPLC method, and the doses of baicalin were selected based on previous reports [25,26,27]. Baicalin is a major constituent in *O. indicum* [18]. Daily treatments were performed for 3 weeks after day 21 (the green bar in Figure 2) at 8:00 a.m., except on the behavioral testing day, mice were administered baicalin 1 h before behavioral testing. The behavioral tests were conducted in the 6th week (the blue bar in Figure 2). Locomotor activity test was initially performed on day 43, followed by TST on day 45. After a 2-day resting period, FST was performed on days 48 and 49 (training phase and testing phase). Afterward, mice were instantly euthanized by giving them 60 mg/kg of thiopental sodium (Anesthal^®^, Jagsonpal Pharmaceutical Ltd., Gurugram, India) i.p., and then blood, frontal cortex, hippocampus, and other necessary organs were collected and kept at −80 °C for neurochemical assessment.

### 2.6. Behavioral Studies

#### 2.6.1. Sucrose Preference Test (SPT)

Anhedonia, one of the major features of depression, is widely indicated via the SPT [28]. The test was performed once a week. Mice were individually placed in a cage for 30 min. After acclimatization, each mouse was presented with 2% sucrose solution for 1 h, and the amount of sucrose intake was recorded. The depressive mice showed a reduction in sucrose consumption.

#### 2.6.2. Locomotor Activity (LA)

Y-maze test was performed to emphasize that none of the drug administrations had an effect on the locomotor function or movement of mice. The Y-maze consisted of three black arms of equal size of 38.5, 3, and 13 cm in length, width, and height, respectively. All arms were oriented at 60° angles from each other. Mouse was placed on one arm and allowed to freely explore for 5 min. After that, the area was cleaned with 70% *w*/*v* of ethanol. The total arm entries were manually recorded.

#### 2.6.3. Tail Suspension Test (TST)

TST is a common behavioral paradigm used to evaluate antidepressant activity. TST was conducted in a dark environment to limit interference. Mice were suspended upside down 30 cm above the ground with their tails attached to a black tape in a position where they could not hold on to the nearby surfaces. The despair behavior was determined by immobility time, which was recorded after 2 min habituation from a 6 min testing period.

#### 2.6.4. Forced Swimming Test (FST)

The FST is one of the most used assessments for antidepressant effects on depressive-like behavior. FST involves the instinctive behavior that occurs when mice are forced to swim in an inescapable cylinder [29]. The mice were individually placed in a glass cylinder (20 cm in diameter, 30 cm in height) containing 15 cm height of water. In a pre-test session, a mouse was forced to swim for 15 min with no observation. Twenty-four hours later (test-session), the mouse was administered a drug 1 h before the test and exposed to the same experimental condition for 5 min. No movement of any legs was considered as immobile or motionless.

### 2.7. Determination of Serum Corticosterone Level

After behavioral assessments, mice were deeply anesthetized with thiopental sodium (Anesthal^®^; 60 mg/kg, i.p.). Approximately half of the total blood volume was collected via cardiac puncture and kept at 20 ± 2 °C for 24 h. Blood was centrifuged to keep only the serum. To investigate the consequence of HPA axis overactivation, corticosterone (CORT) level was measured by using corticosterone ELISA kit (Abcam, Cambridge, UK). An amount of 25 µL of biotinylated corticosterone protein (BCP) was added to 25 µL of mouse serum to bind with corticosterone. Then, the plate was incubated at room temperature for 2 h and washed with 200 µL of washing solution 5 times. Then, 50 µL of streptavidin–peroxidase (SP) conjugate was pipetted into the well to attach to the BCP-corticosterone complex, and the reaction was allowed to occur for 30 min. After cleaning with a washing solution, 50 µL chromogen was added to each well. After 20 min of incubation, a blue color was observed. The reaction was terminated by adding stop solution, and the blue color changed to a yellow color, which was detected at 450 nm. The detector was a microplate reader (PerkinElmer, Inc., Shelton, CT, USA). Afterward, the concentration of serum corticosterone was calculated by using Gaindata^®^ (arigo, Taiwan).

### 2.8. Quantitative Real-Time Polymerase Chain Reaction (qPCR)

qPCR was applied to investigate the mechanism of the *OIS* extract on multifactorial etiology of depression. Total RNA was isolated from the hippocampus and the frontal cortex by using TRIzol^®^ reagent (Invitrogen^TM^, Thermo Fisher Scientific, Waltham, MA, USA), and then chloroform was added for phase separation. RNA was solubilized in the aqueous phase and then precipitated via isopropanol. Nuclease-free water was used in order to dissolve the pellet. Oligo(dT) 12–18 primer, accompanied by deoxynucleotide triphosphate (dNTP), was used for complementary DNA (cDNA) synthesis with Superscript^TM^ III reverse transcriptase (Invitrogen^TM^, Thermo Fisher Scientific, Waltham, MA, USA) via FlexCycler^2^ PCR Thermal Cycler (Analytik Jena, Jena, Germany). Gene-related primer (Pacific Science, Thailand) was attached to single-stranded DNA (ssDNA) after denaturation process in the individually optimal temperature (annealing step) to start extension. During the extension, double-stranded DNA (dsDNA) was measured using the non-specific fluorescent dye method, SYBR^®^ green supermixes (Bio-Rad, Hercules, CA, USA). All steps were performed in Bio-Rad CFX96 Touch Real-Time PCR Detection System (Bio-Rad, Hercules, CA, USA). The amount of fluorescence in a sample was plotted against the cycle number, and the quantitation was recorded as Ct value evaluated using Bio-Rad CFX96 analysis ver. 3.1 software. The target gene expressions were expressed in fold difference relative, and glyceraldehyde-3-phosphate dehydrogenase (GAPDH) was used as a reference gene. The fold difference relatives were calculated by using the 2^−∆∆Ct^ method. All primers were synthesized via Macrogen (Seoul, South Republic of Korea), and they are presented in Table 1.

### 2.9. High-Performance Liquid Chromatography (HPLC) Analysis and Method Validation

The *OIS* extract was analyzed via a reversed-phase HPLC system using a Hypersil ODS column (Agilent Technologies Inc., Santa Clara, CA, USA; 4 × 250 mm, 5 µm) with a gradient elution with 30–70% methanol and 0.2% formic acid in ultrapure water with a flow rate of 1.2 mL/min. The signal intensity was detected at 254 and 275 nm during a 60 min cycle time. Standard baicalin, quercetin, kaempferol, baicalein, chrysin, and oroxylin A were used in this HPLC analysis. In order to quantify the chemical constituents, 10 mg of the *OIS* extract was dissolved in methanol to make a stock solution (10 mg/mL) and diluted to several appropriate solutions using mobile phase for quantification of chemical constituents. The extract solution was filtered through a 0.45 µm nylon syringe filter before injection. The amount of baicalin was determined by using standard curves (1, 2, 3, 4, 5, and 6 µg/mL), and the others (baicalein, chrysin, and oroxylin A) were determined by using standard curves (2.5, 5, 7.5, 10, 12.5, and 15 µg/mL), which were prepared from a stock solution (1 mg/mL). HPLC validations are classified according to ICH guidelines (ICH Guidelines, 1996).

### 2.10. Statistical Analysis

Non-stress group and stress-induced group were analyzed using paired Student’s *t*-test. One-way analysis of variance (ANOVA) was performed, followed by the Tukey test for multiple comparisons among the treatment groups. One-way repeated ANOVA was used for the assessment of sucrose intake and training for MWM in each mouse. The significant difference was determined with *p* < 0.05. Results are expressed as mean ± standard error of mean (S.E.M), except for in vitro studies, which are expressed as mean ± standard deviation (SD). All data were analyzed using Sigma Stat^®^ ver. 3.5 software (SYSTAT Software Inc., San Jose, CA, USA).

## 3. Results

### 3.1. Inhibitory Activity of the OIS Extract on MAO-A and MAO-B Enzymes

MAO-A is responsible for the metabolism of the neurotransmitters that are related to depression. The amount of fluorescent 4-hydroxyquinolone showed a direct correlation with MAO-A activity; thus, its fluorescent intensity was used to calculate the IC_50_ value by varying the concentration of the compound of interest or the extract. The results showed that the *OIS* extract exhibited an inhibitory effect against the MAO-A and MAO-B enzymes with IC_50_ values of 36.07 ± 0.4704 µg/mL and 146.8 ± 4.939 µg/mL, respectively. The results also confirmed that the *OIS* extract has more selectivity to the MAO-A isoform than to the MAO-B isoform, as shown in Table 2.

### 3.2. Effect of the OIS Extract on Anhedonia in Mice Subjected to UCMS

**Figure 3:**
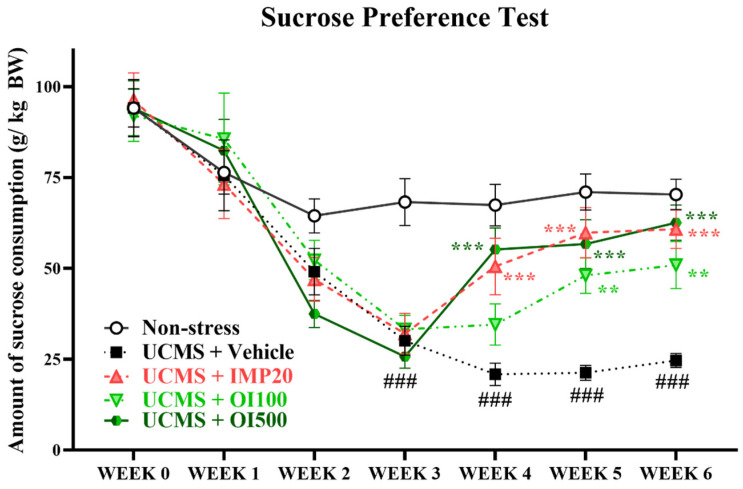
The *OIS* extract (100 and 500 mg/kg) significantly improved sucrose preference, which was diminished by long-term stress. SPT was performed every week to monitor anhedonia. The results are expressed as mean ± S.E.M. (*n* = 10 per each group). ^###^ *p* < 0.001 vs. the non-stress group. ** *p* < 0.01 and *** *p* < 0.001 vs. the vehicle-treated UCMS group.

Anhedonia, a main symptom of depression, is the inability to feel pleasure from daily activities. This manifestation is commonly indicated by the sucrose intake in an animal model of depression [28]. The UCMS-induced group that was treated with the vehicle showed a significant reduction in sucrose consumption after 3 weeks of stress exposure compared to the non-stress group (t[18] = 5.061, *p* < 0.001). Treatment with the *OIS* extract at a dose of 500 mg/kg for one week showed a significant improvement in sucrose intake similar to the treatment with imipramine, a well-known antidepressant (F[3,36] = 7.192). Similarly, treatment with the *OIS* extract at a dose of 100 mg/kg ameliorated the impaired sucrose consumption after 2 weeks (F[3,36] = 10.134). The results are shown in Figure 3.

### 3.3. Effect of the OIS Extract on Despair Behavior Induced by UCMS and Locomotor Activity

**Figure 4:**
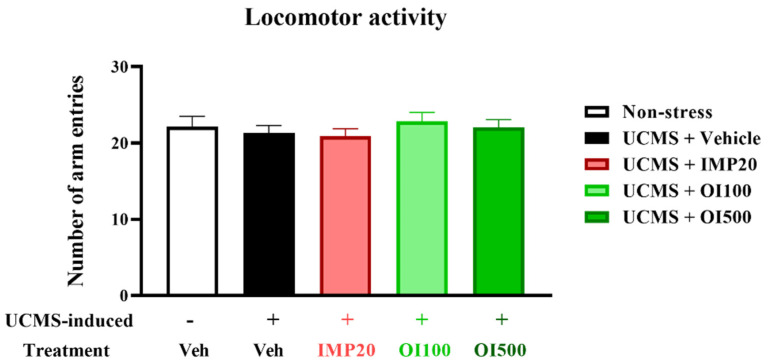
The *OIS* extract (100 and 500 mg/kg) showed no effect on locomotor function in UCMS-induced mice, as determined using the Y-maze test. The data were analyzed from number of arm entries and expressed as mean ± S.E.M. (*n* = 10 per each group).

**Figure 5:**
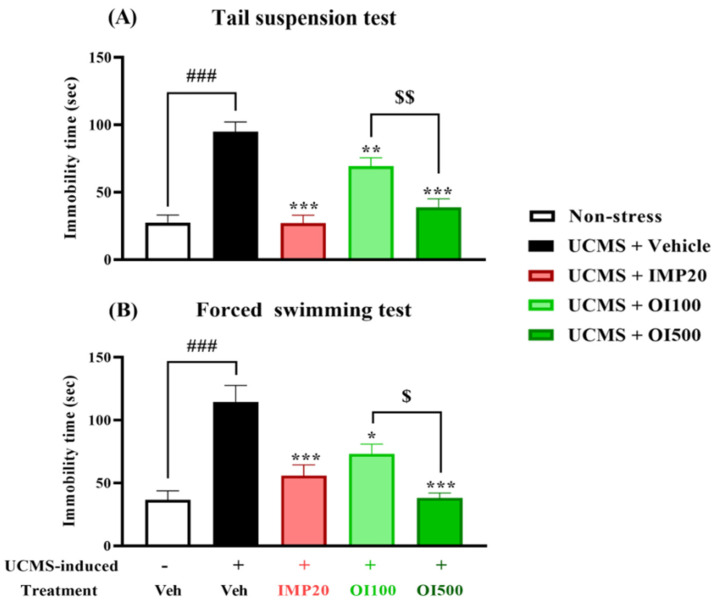
The *OIS* extract (100 and 500 mg/kg) significantly decreased the immobility time, which was an implication for despair behavior in TST (**A**) and FST (**B**). Behavioral tests were performed after treatment for 3 weeks. The results are expressed as mean ± S.E.M. (*n* = 10 per each group). ^###^ *p* < 0.001 vs. the non-stress group. * *p* < 0.05, ** *p* < 0.01, and *** *p* < 0.001 vs. the vehicle-treated UCMS group. ^$^ *p* < 0.05 and ^$$^ *p* < 0.01 compared with different doses of the *OIS* extract.

In order to exclude the effect of the *OIS* extract on mouse locomotor activity, the Y-maze task was performed to analyze the locomotor function in each mouse. There was no significant difference among the groups, as shown in Figure 4, indicating that neither imipramine nor the *OIS* extract exerted an effect on the locomotor activity in the UCMS mice. A behavioral state of despair, as observed in human depression, is determined by immobility in the TST and FST using a mouse model. Both tests are commonly used to measure stress-induced behavior in mice [31]. The group that was treated with the vehicle after being exposed to stress showed a significant increment in the immobility time in both the TST and FST (t[18] = −7.452 in the TST and t[18] = −5.186 in the FST, respectively, *p* < 0.001) when compared to the control group (Figure 5). The *OIS*-extract-treated UCMS group significantly reduced the immobility time compared with the vehicle-treated UCMS group (F[3,36] = 23.118 in the TST and F[3,36] = 13.250 in the FST), similar to the group that was treated with imipramine, a reference antidepressant. In addition, the *OIS* extract exhibited an antidepressant effect in a dose-dependent manner, as shown in Figure 5. Taken together, *OIS* extract and imipramine exert antidepressant activity without any effect on the locomotor activity.

### 3.4. Effect of the OIS Extract on Serum Corticosterone (CORT) Level in UCMS-Induced Mice

**Figure 6:**
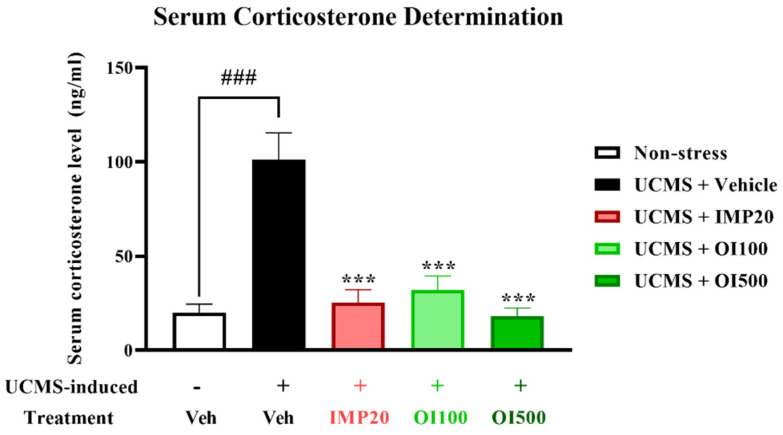
The *OIS* extract (100 and 500 mg/kg) and imipramine (20 mg/kg) reduced the elevation in the serum corticosterone level induced by the UCMS. The data are expressed as mean ± S.E.M. (*n* = 5). ^###^ *p* < 0.001 vs. the non-stress group. *** *p* < 0.001 vs. the vehicle-treated UCMS group.

The hypersecretion of GCs, which can be induced by an exposure to long-term stress, also contributes to the development of depression [32]. After an exposure to chronic stress, the serum corticosterone levels were significantly increased in the mice that were treated with the vehicle when compared to the non-stress group ((t[8] = −5.415, *p* < 0.001). However, treatment with imipramine (20 mg/kg) and the *OIS* extract (100 and 500 mg/kg) ameliorated the oversecretion of corticosterone levels in the UCMS-induced mice (F[3,16] = 18.231) (Figure 6).

### 3.5. The OIS Extract Normalized HPA Axis Overactivation and Neurogenesis via Changes in FKBP5, GR, SGK-1, BDNF, and CREB mRNA Expressions in Affected Brains

**Figure 7:**
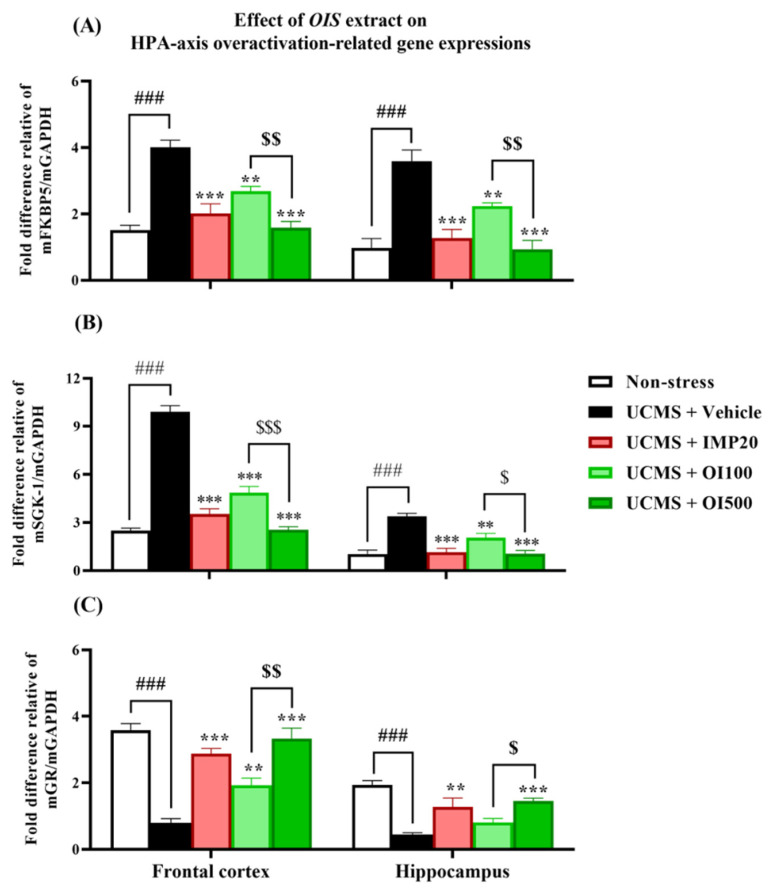
The *OIS* extract (100 and 500 mg/kg) and imipramine (20 mg/kg) alleviated the impaired regulation of the HPA axis by decreasing FKBP5 (**A**) and SGK-1 (**B**) mRNA expressions, while improving GR mRNA expression (**C**) in both affected brain areas. The results are expressed as mean ± S.E.M. (*n* = 6). ^###^ *p* < 0.001 vs. the non-stress group. ** *p* < 0.01 and *** *p* < 0.001 vs. the vehicle-treated UCMS group. ^$^ *p* < 0.05, ^$$^ *p* < 0.01, and ^$$$^ *p* < 0.001 compared with the different doses of the *OIS* extract.

**Figure 8:**
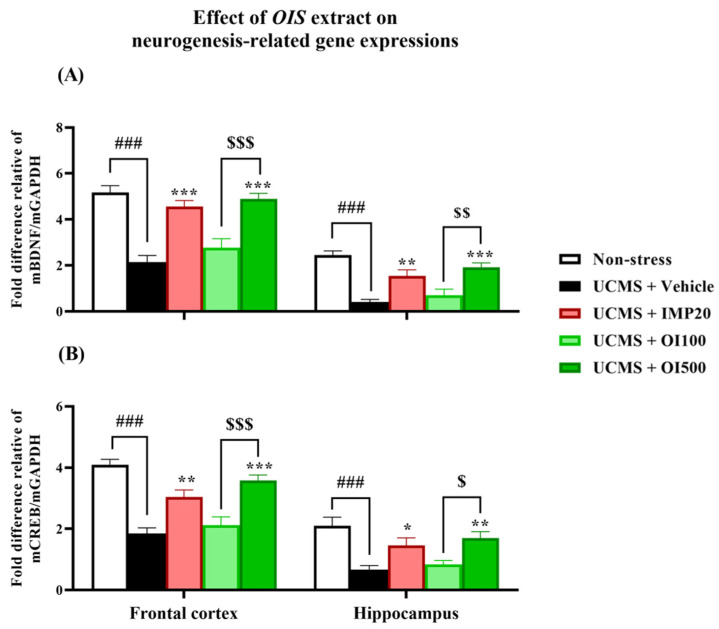
The *OIS* extract in the dose of 500 mg/kg normalized the depletion of neurogenesis and neuroplasticity induced by chronic stress which was determined by mRNA expressions of BDNF (**A**) and CREB (**B**) in the same manner as imipramine. The results are expressed as mean ± S.E.M. (*n* = 6). ^###^ *p* < 0.001 vs. the non-stress group. * *p* < 0.05, ** *p* < 0.01 and *** *p* < 0.001 vs. the vehicle-treated UCMS group. ^$^ *p* < 0.05, ^$$^ *p* < 0.01 and ^$$$^ *p* < 0.001 compared with different doses of the *OIS* extract.

The overactivation of the HPA axis and impaired neurogenesis are involved in the pathogenesis of depression [8]. Therefore, the mRNA expressions of the related genes were investigated. As shown in Figure 7 and Figure 8, the mRNA expressions of FKBP5 (t[10] = −9.425 and −5.868 in FC and HP, respectively; *p* < 0.001) and SGK-1 (t[10] = −17.690 and −2.353 in FC and HP; *p* < 0.001) were markedly increased, while the mRNA expressions of GR (t[10] = 11.873 and 10.531 in FC and HP; *p* < 0.001), BDNF (t[10] = 9.911 and 9.214 in FC and HP; *p* < 0.001), and CREB (t[10] = 8.993 and 4.649 in FC and HP; *p* < 0.001) were significantly decreased in both impacted brain regions of the mice that were subjected to UCMS. After daily treatment with the *OIS* extract (100 and 500 mg/kg), the upregulated mRNA expressions of FKBP5 and SGK-1 were alleviated (F[3,20] = 23.573 and 93.881 in the frontal cortex, and F[3,20] = 21.625 and 22.281 in the hippocampus, respectively), whereas the impaired expression of GR was normalized (F[3,20] = 27.307 and 8.894) in the frontal cortex and in the hippocampus in a dose-dependent manner, which is similar to the positive control, imipramine. On the other hand, only the treatment with the *OIS* extract at a dose of 500 mg/kg could alter the gene expressions of BDNF and CREB (F[3,20] = 27.347 and 13.720 in the frontal cortex and F[3,20] = 10.661 and 7.058 in the hippocampus, respectively).

### 3.6. HPLC Analysis of the OIS Extract and Method Validation

Baicalin (1), quercetin (2), kaempferol (3), baicalein (4), chrysin (5), and oroxylin A (6) were used as markers in the HPLC analysis of the *OIS* extract. From the method validation, it could be concluded that the selected method was reliable and suitable to quantify all of the compounds that were present in the *OIS* extract. Precision from within days and between days had percentage relative standard deviations (%RSDs) of less than 3%, and the linearity showed a high correlation between the peak area and concentration (R^2^ > 0.999). The accuracy, as determined by the percentage recovery of spiked standards, was always between 99% and 105%. From all validation parameters, it was found that this analytical method was reliable and could be used for the analysis of the six compounds.

**Figure 9:**
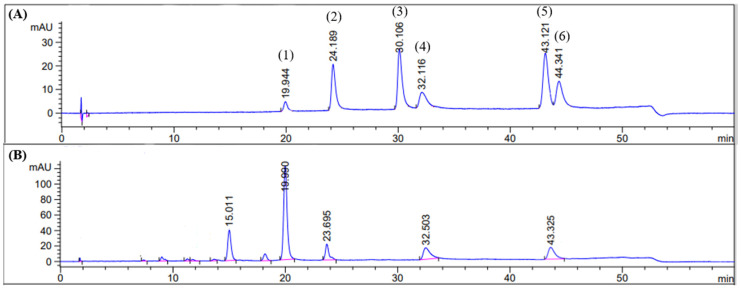
Chromatogram of standard: baicalin (1), quercetin (2), kaempferol (3), baicalein (4), chrysin (5), and oroxylin A (6), respectively (**A**), and the *OIS* extract (**B**). Detection wavelength was 275 nm.

HPLC was further used to measure the amount of compounds in the *OIS* extract, and the retention times shown in Figure 9 were consistent with those of the standards. With this HPLC analysis, three compounds in the *OIS* extract were identified, viz. baicalin (84.22 ± 0.22 mg/g extract), baicalein (23.45 ± 0.11 mg/g extract), and chrysin (11.38 ± 0.09 mg/g extract), while the peaks with the retention times of 15.011 and 23.695 were not identified.

## 4. Discussion

MDD is prevalent among people of many ages around the world, causing a serious negative impact. Although various types of antidepressants are available, approximately 10–30% of patients show partial responses, along with a poor quality of life or even a high relapse tendency [33]. Moreover, an early state of MDD, called “minor depression”, needs to be concerned. Minor depression shows similar symptoms to MDD and can become severe depression in one to three years if there is no diagnosis or treatment [34]. There is strong evidence showing that alternative treatments, such as psychotherapy or herbal medicine, can have significant and beneficial effects on minor depression and prevent the onset of MDD, with lower side effects and lower costs compared with synthetic antidepressants [35]. For this reason, *OIS* extract was investigated for its potential as an herbal antidepressant.

First, the *OIS* extract was preliminarily screened for its inhibitory effect on the MAO-A enzyme, a specific isoform of MAOs, which mainly metabolize monoamine neurotransmitters, including noradrenaline and 5-hydroxytryptamine. MAO-A has been accepted to be the target for antidepressant development [36]. In the present investigation, we demonstrated that the *OIS* extract exhibited a more selective inhibitory effect to MAO-A than to MAO-B. Consistent with previous findings, Zhu et al. demonstrated that the antidepressant effect of baicalin in animal models of depression were related to the inhibitory activity of MAO, especially to that of MAO-A [37]. Baicalein and its aglycone part are the active constituents in *OIS* extract. This finding suggests that the inhibitory effect on MAO-A may be involved in the antidepressant activity of *OIS* extract.

UCMS, a well-established study used to evaluate the antidepressant effects of novel substances, was used in the experiment since it does not only induce depressive-related behaviors, but also causes physiological and neurological changes, which are associated with clinical depression [38]. The UCMS-treated mice exhibited a significant reduction in the sucrose FST and TST. Both tests used the same rationale, i.e., immobility is considered as a resignation to a state of despair in which the mouse learned that there was no escape from the situation. In the non-stress group, the mice showed signs of struggle by trying to climb the glass cylinder or by moving their legs. On the other hand, the UCMS group expressed a significant longer duration of immobility compared to the non-stress group. The results showed that the *OIS* extract markedly alleviated hopeless behaviors by reducing the immobility time in both tests. To ensure that the results were only obtained from the antidepressant effect of the *OIS* extract, the Y-maze task was used to evaluate the locomotor activity. The test showed that both imipramine and the *OIS* extract had no effects on the locomotor function. Taken together, the *OIS* extract exhibited a potential to be an antidepressant agent in a chronic stress model of depression in a mouse.

Induction via UCMS could cause physiological changes, such as hypercortisolism, which is associated with the HPA axis hyperfunction. This condition plays an important role in the pathogenesis of depression [3]. The serum corticosterone level was determined via ELISA to verify this hypothesis. The result showed that the serum corticosterone level was significantly increased in the stress-induced group with the vehicle treatment compared to the non-stress group. Treatment with the *OIS* extract attenuated the level of corticosterone similar to the effect of imipramine. In addition, the mRNA expressions of gene-related HPA axis regulation were investigated in order to confirm the mechanism of the *OIS* extract. FKBP5, a competitive inhibitor of GCs in GR binding, exerts a negative effect on the HPA axis regulation. FKBP5 can cause a resistance to GR, leading to impaired negative feedback and resulting in the hypersecretion of GCs. Patients with depressive symptoms showed a higher expression of FKPB5, but this can be reversed with a long-term antidepressant treatment [39]. Consistently, in this study, the chronic stress-exposed group showed a significantly higher expression of FKBP5 compared to the control group, especially in the depressive-related brain areas, viz. the hippocampus and the frontal cortex. The daily administration of the *OIS* extract and imipramine could attenuate the elevation of FKPB5 expression in the affected brain area. Moreover, it is suggested that hypercortisolism secondarily downregulated the GR expression [7]. The reduction in available GRs to bind with GCs can lead to an impairment in negative feedback. Our finding indicated that the GR mRNA expression significantly decreased in the UCMS group, which could be improved after treatment with the *OIS* extract. SGK-1 is one of the brain proteins, which is transcriptionally regulated by GCs, that also participates in neuronal proliferation and apoptosis. A GC elevation after the exposure to chronic stress can cause an increment in SGK-1 in the hippocampus, leading to decreased hippocampus and frontal cortex neurogenesis [40]. Consistent with previous findings, UCMS caused an apparent increase in the SGK-1 expression, and treatment with the *OIS* extract for at least three weeks ameliorated this abnormality. Furthermore, there is a linkage between SGK-1 and BDNF, a neurotrophin that has a leading role in the etiology of depression [9]. Although the molecular mechanism of their interaction is still unclear, it is known that GR overactivation can suppress the BDNF signaling pathway, leading to neuroplasticity impairment [41]. BDNF and its receptor, tropomyosin kinase receptor B (TrkB), are regulators of dendritic growth in the central nervous system (CNS). The transcription of BDNF/TrkB signaling needs a transcription factor, named cyclic adenosine monophosphate (cAMP) response element-binding protein or CREB. It is well known that lowering CREB expression can decrease the level of BDNF [42]. Patients with depressive states express reductions in BDNF and CREB in the hippocampus, and the neurogenesis and neuroplasticity can be improved after treatment with an antidepressant. Consequently, we found that the UCMS paradigm could impair the mRNA expressions of BDNF and CREB in the mouse brain, and treatment with the *OIS* extract in the UCMS mice improved the neurogenesis.

**Figure 10:**
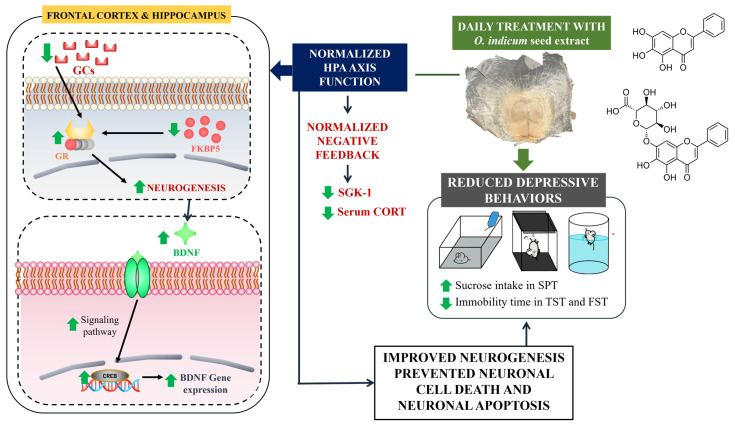
The possible molecular mechanisms of *OIS* extract.

Lastly, a phytochemical analysis of the *OIS* extract via HPLC was able to identify and quantify, among five major components, the three flavonoids, including baicalin (84.22 ± 0.22 mg/g extract), baicalein (23.45 ± 0.11 mg/g extract), and chrysin (11.38 ± 0.089 mg/g extract). Many studies confirmed that all of these flavonoids could alleviate depressive symptoms with various mechanisms of action. It was proven that a high level of GC from chronic CORT injection could reduce the available GR in the cytoplasm and increase the GR in the nucleus, resulting in an impairment in the negative feedback [43]. However, treatment with baicalin (40, 80, and 160 mg/kg) significantly alleviated a depressive-related manifestation in the CORT-induced mice via the normalization of the GR function through SGK1- and FKBP5-mediated GR phosphorylation. In addition, baicalin also ameliorated UCMS-induced depressive-like behaviors through the hippocampal BDNF/ERK/CREB signaling pathway in male ICR mice [26]. Interestingly, an investigation of the antidepressant activity of baicalein has shown that the chronic treatment of baicalein (4 mg/kg/day, i.p.) for 21 days also reduced the immobility time (FST and TST) in the UCMS rat model. The possible mechanism of baicalein is at least partly mediated via ERK-mediated neurogenesis in the hippocampus [44]. Moreover, the previous work reported that chrysin at doses of 5 and 20 mg/kg could attenuate depressive behaviors resulting from bilateral olfactory bulbectomy in mice by preventing the reduction in hippocampal BDNF and 5-HT, and it also ameliorated HPA axis dysregulation by reducing the serum CORT level and related regulatory hormones (CRH and ACTH) in the chronically stressed mice [45,46]. Taken together, the antidepressant effect of *OIS* extract could be explained by the synergistic effects of its major constituents with multiple molecular mechanisms, as shown in Figure 10.

## 5. Conclusions

*OIS* extract possesses multiple modes of action in UCMS-induced depressive mice, such as the amelioration of anhedonia and despair behavior. These effects could be attributed to its bioactive flavones, baicalin and baicalein. The obtained results emphasize the potential of *OIS* extract as an effective and novel alternative treatment for MDD. However, further investigations are still needed to deepen our understanding and knowledge of the safety profile, contraindications, interactions with other drugs, and fine molecular mechanisms underlying the beneficial effects of *OIS* extract. Particularly, this herbal plant may also act through yet unexplored biological pathways that are implicated in various mental disorders.

## Figures and Tables

**Table 1:** Summary of primer sequences used for qPCR.

Groups	Genes	Primer Sequences	Product Length	Reference
House-keeping gene	GAPDH	Forward: 5′-ACC ACA GTC CAT GCC ATC AC-3′ Reverse: 5′-TCC ACC ACC CTG TTG CTG TA-3′	452 bp	[30]
Neurogenesis	BDNF	Forward: 5′-GAC AAG GCA ACT TGG CCT AC-3′ Reverse: 5′-CCT GTC ACA CAC GCT CAG CTC-3′	334 bp	[30]
	CREB	Forward: 5′-TAC CCA GGG AGG AGG AAT AC-3′ Reverse: 5′-GAG GCT GCT TGA ACA ACA AC-3′	183 bp	[30]
HPA axis	SGK-1	Forward: 5′-GGG TGC CAA GGA TGA CTT TA-3′ Reverse: 5′-CTC GGT AAA CTC GGG ATA GA-3′	154 bp	[30]
	GR	Forward: 5′- CAC TAA TCC TCT CCA TCC TAC-3′ Reverse: 5′- AAT GTC TGC TGC CTT CTG-3′	479 bp	[25]
	FKBP5	Forward: 5′- GAA CCC AAT GCT GAG CTT ATG-3′ Reverse: 5′- ATG TAC TTG CCT CCC TTG AAG-3′	149 bp	[5]

**Table 2:** Inhibitory effect of the *OIS* extract on MAO-A activity compared with clorgyline and deprenyl, which are selective inhibitors for MAO-A and MAO-B, respectively.

Extract/Substance	Inhibition of MAO-A Enzyme	Inhibition of MAO-B Enzyme
	IC_50_ (µg/mL for Extract and µM for Compound)	IC_50_ (µg/mL for Extract and µM for Compound)
*OIS* extract	36.07 ± 0.47	146.80 ± 4.94
Clorgyline	0.001201 ± 0.000014	20.38 ± 0.33
Deprenyl	10.49 ± 0.11	0.052 ± 0.009

Data are expressed as mean ± S.D.

## Data Availability

The data used to support the findings of this study can be made available by the corresponding authors upon request.

## Supplementary Materials

The following supporting information can be downloaded at: https://www.mdpi.com/article/10.3390/nu15224742/s1. Figure S1. Inhibitory effect of *O. indicum* seed on MAO-A and MAO-B (panel A and B, respectively). The inhibition graph was plot between log(concentration) (X-axis) and %inhibition (Y-axis). Figure S2. Inhibitory effect of Clorgyline on MAO-A and MAO-B (panel A and B, respectively). The inhibition graph was plot between log(concentration) (X-axis) and %inhibition (Y-axis). Figure S3. Inhibitory effect of Deprenyl on MAO-A and MAO-B (panel A and B, respectively). The inhibition graph was plot between log(concentration) (X-axis) and %inhibition (Y-axis). Table S1. The preliminarily screening of *O. indicum* parts by using HPLC analysis. Table S2. Paired Student’s *t*-test and One-way analysis of variance (ANOVA) test of SPT. Table S3. Paired Student’s *t*-test and One-way analysis of variance (ANOVA) test of TST. Table S4. Paired Student’s *t*-test and One-way analysis of variance (ANOVA) test of FST. Table S5. Paired Student’s *t*-test and One-way analysis of variance (ANOVA) test of serum CORT level. Table S6. Paired Student’s *t*-test and One-way analysis of variance (ANOVA) test of FK506 binding protein 51 (FKBP5) in frontal cortex and hippocampus. Table S7. Paired Student’s *t*-test and One-way analysis of variance (ANOVA) test of serine/threonine-protein kinase 1 (SGK-1) in frontal cortex and hippocampus. Table S8. Paired Student’s *t*-test and One-way analysis of variance (ANOVA) test of glucocorticoid receptor (GR) in frontal cortex and hippocampus. Table S9. Paired Student’s *t*-test and One-way analysis of variance (ANOVA) test of brain-derived neurotrophic factor (BDNF) in frontal cortex and hippocampus. Table S10. Paired Student’s *t*-test and One-way analysis of variance (ANOVA) test of cyclic AMP-responsive element-binding protein (CREB) in frontal cortex and hippocampus. Table S11. Validation result of the HPLC method for determination of baicalin, baicalein, chrysin, and oroxylin A.
