# Supplementary material for: Antidepressant-like Effect of Oroxylum indicum Seed Extract in Mice Model of Unpredictable Chronic Mild Stress
Source: Nutrients. 2023 Nov 10;15(22):4742. doi: 10.3390/nu15224742 (PMC10675042; doi:10.3390/nu15224742)
Supplement: Supplementary file 1 [file nutrients-15-04742-s001.zip › nutrients-2699229-supplementary.pdf]

Supplementary Materials

Antidepressant-like effect of *Oroxylum indicum* Seed Extract in Mice Model of Unpredictable Chronic Mild Stress

Chorpeth Chalermwongkul[1], Charinya Khamphukdee[2], Juthamart Maneenet[3], Supawadee Daodee[3], Orawan Monthakantirat[3], Chantana Boonyarat[3], Yutthana Chotritthirong[1], Suresh Awale[4], Anake Kijjoa[2,5], Yaowared Chulikhit[3]*

[1] Graduated School of Pharmaceutical Sciences, Khon Kaen University, Khon Kaen, 40002, Thailand; c.chorpeth@kkumail.com, yutthana_ch@kkumail.com
[2] Division of Pharmacognosy and Toxicology, Faculty of Pharmaceutical Sciences, Khon Kaen University, Khon Kaen 40002, Thailand; charkh@kku.ac.th
[3] Division of Pharmaceutical Chemistry, Faculty of Pharmaceutical Sciences, Khon Kaen University, Khon Kaen, 40002, Thailand; juthamart_pp@hotmail.com, csupawad@kku.ac.th, oramon@kku.ac.th, chaboo@kku.ac.th
[4] Natural Drug Discovery Laboratory, Institute of Natural Medicine, University of Toyama, 2630 Sugitani, Toyama, 930-0154, Japan.; suresh@inm.u-toyama.ac.jp
[5] ICBAS-Instituto de Ciências Biomédicas Abel Salazar and CIIMAR, Universidade do Porto, Rua de Jorge Viterbo Ferreira 228, 4050-313 Porto, Portugal; ankijjoa@icbas.up.pt
* Correspondence: yaosum@kku.ac.th; Tel.: +66-81-3802357

Table S1. The preliminarily screening of *O. indicum* parts by using HPLC analysis.

**Compounds**	**Amount (mg/g extract)**		
	**Seed**	**Pod**	**Root**
**Baicalin**	84.22 ± 0.22	24.07 ± 0.19	10.82 ± 0.019
**Baicalein**	23.45 ± 0.11	21.04 ± 0.25	43.60 ± 0.042
**Chrysin**	11.38 ± 0.089	9.18 ± 0.053	9.73 ± 0.079
**Oroxylin A**	ND	16.07 ± 0.053	35.60 ± 0.12

Statistical Analysis of Effect of the OIS extract on UCMS-Induced Anhedonia Behavior Using Sucrose Preference Test (SPT)

Table S2. Paired Student's *t*-test and One-way analysis of variance (ANOVA) test of SPT

**Group comparison**	**ANOVA followed by Tukey's post hoc test**	
	P	**$F(DF_{between\ group}, DF_{residual})$**
**Week 0**		
non-stress group vs. UCMS + vehicle group	0.994	t(18) = -0.00752
All UCMS-induced groups	0.980	F(3,36) = 0.0619
**Week 1**		
non-stress group vs. UCMS + vehicle group	0.946	t(18) = 0.0690
All UCMS-induced groups	0.812	F(3,36) = 0.318
**Week 2**		
non-stress group vs. UCMS + vehicle group	0.069	t(18) = 1.934
All UCMS-induced groups	0.274	F(3,36) = 1.347
**Week 3**		
non-stress group vs. UCMS + vehicle group	< 0.001	t(18) = 5.016
All UCMS-induced groups	0.613	F(3,36) = 0.609
**Week 4**		
non-stress group vs. UCMS + vehicle group	< 0.001	t(18) = 7.168
All UCMS-induced groups	< 0.001	F(3,36) = 7.192
UMCS + vehicle group vs. UCMS + IMP20 group	0.005	
UMCS + vehicle group vs. UCMS + OIS100 group	0.233	
UMCS + vehicle group vs. UCMS + OIS500 group	0.001	
UCMS + OIS100 group vs. UCMS + OIS500 group	0.078	
**Week 5**		
non-stress group vs. UCMS + vehicle group	< 0.001	t(18) = 9.310
All UCMS-induced groups	< 0.001	F(3,36) = 10.134
UMCS + vehicle group vs. UCMS + IMP20 group	< 0.001	
UMCS + vehicle group vs. UCMS + OIS100 group	0.008	
UMCS + vehicle group vs. UCMS + OIS500 group	< 0.001	
UCMS + OIS100 group vs. UCMS + OIS500 group	0.685	
**Week 6**		
non-stress group vs. UCMS + vehicle group	< 0.001	t(18) = 9.960
All UCMS-induced groups	< 0.001	F(3,36) = 12.664
UMCS + vehicle group vs. UCMS + IMP20 group	< 0.001	
UMCS + vehicle group vs. UCMS + OIS100 group	0.003	
UMCS + vehicle group vs. UCMS + OIS500 group	< 0.001	
UCMS + OIS100 group vs. UCMS + OIS500 group	0.351	

Statistical Analysis of Effect of the OIS extract on UCMS-Induced Despair Behaviors Using Tail Suspension Test (TST) and Forced Swimming Test (FST)

Table S3. Paired Student's *t*-test and One-way analysis of variance (ANOVA) test of TST

**Group comparison**	**ANOVA followed by Tukey's post hoc test**	
	P	**F($DF_{between\ group}$, $DF_{residual}$)**
non-stress group vs. UCMS + vehicle group	< 0.001	t(18) = -7.452
All UCMS-induced groups	< 0.001	F(3,36) = 23.118
UMCS + vehicle group vs. UCMS + IMP20 group	< 0.001	
UMCS + vehicle group vs. UCMS + OIS100 group	0.035	
UMCS + vehicle group vs. UCMS + OIS500 group	< 0.001	
UCMS + OIS100 group vs. UCMS + OIS500 group	0.009	

Table S4. Paired Student's *t*-test and One-way analysis of variance (ANOVA) test of FST

**Group comparison**	**ANOVA followed by Tukey's post hoc test**	
	P	**F($DF_{between\ group}$, $DF_{residual}$)**
non-stress group vs. UCMS + vehicle group	< 0.001	t(18) = -5.186
All UCMS-induced groups	< 0.001	F(3,36) = 13.250
UMCS + vehicle group vs. UCMS + IMP20 group	< 0.001	
UMCS + vehicle group vs. UCMS + OIS100 group	0.013	
UMCS + vehicle group vs. UCMS + OIS500 group	< 0.001	
UCMS + OIS100 group vs. UCMS + OIS500 group	0.043	

Statistical Analysis of Effect of the OIS extract on UCMS-Induced Hypersecretion of Glucocorticoids Using Serum Corticosterone (CORT) Level

Table S5. Paired Student's *t*-test and One-way analysis of variance (ANOVA) test of serum CORT level

**Group comparison**	**ANOVA followed by Tukey's post hoc test**	
	P	**F($DF_{between\ group}$, $DF_{residual}$)**
non-stress group vs. UCMS + vehicle group	< 0.001	t(18) = -5.415
All UCMS-induced groups	< 0.001	F(3,16) = 18.231
UMCS + vehicle group vs. UCMS + IMP20 group	< 0.001	
UMCS + vehicle group vs. UCMS + OIS100 group	< 0.001	
UMCS + vehicle group vs. UCMS + OIS500 group	< 0.001	
UCMS + OIS100 group vs. UCMS + OIS500 group	0.695	

Statistical Analysis of Effect of the OIS extract on UCMS-Induced Hyperactivation of Hypothalamic-pituitary-adrenal (HPA) axis in Frontal Cortex and Hippocampus Using Quantitative real-time polymerase chain reaction (qPCR)

Table S6. Paired Student's *t*-test and One-way analysis of variance (ANOVA) test of FK506 binding protein 51 (FKBP5) in frontal cortex and hippocampus

**Group comparison**	**ANOVA followed by Tukey's post hoc test**	
	P	**F($DF_{between\ group}$, $DF_{residual}$)**
**Frontal cortex**		
non-stress group vs. UCMS + vehicle group	< 0.001	t(10) = -9.425
All UCMS-induced groups	< 0.001	F(3,20) = 23.573
UMCS + vehicle group vs. UCMS + IMP20 group	< 0.001	
UMCS + vehicle group vs. UCMS + OIS100 group	0.002	
UMCS + vehicle group vs. UCMS + OIS500 group	< 0.001	
UCMS + OIS100 group vs. UCMS + OIS500 group	0.009	
**Hippocampus**		
non-stress group vs. UCMS + vehicle group	< 0.001	t(10) = -5.868
All UCMS-induced groups	< 0.001	F(3,20) = 21.625
UMCS + vehicle group vs. UCMS + IMP20 group	< 0.001	
UMCS + vehicle group vs. UCMS + OIS100 group	0.006	
UMCS + vehicle group vs. UCMS + OIS500 group	< 0.001	
UCMS + OIS100 group vs. UCMS + OIS500 group	0.009	

Table S7. Paired Student's *t*-test and One-way analysis of variance (ANOVA) test of serine/threonine-protein kinase 1 (SGK-1) in frontal cortex and hippocampus

**Group comparison**	**ANOVA followed by Tukey's post hoc test**	
	P	**F($DF_{between\ group}$, $DF_{residual}$)**
**Frontal cortex**		
non-stress group vs. UCMS + vehicle group	< 0.001	t(10) = -17.690
All UCMS-induced groups	< 0.001	F(3,20) = 93.881
UMCS + vehicle group vs. UCMS + IMP20 group	< 0.001	
UMCS + vehicle group vs. UCMS + OIS100 group	< 0.001	
UMCS + vehicle group vs. UCMS + OIS500 group	< 0.001	
UCMS + OIS100 group vs. UCMS + OIS500 group	<0.001	
**Hippocampus**		
non-stress group vs. UCMS + vehicle group	< 0.001	t(10) = -2.353
All UCMS-induced groups	< 0.001	F(3,20) = 21.625
UMCS + vehicle group vs. UCMS + IMP20 group	< 0.001	
UMCS + vehicle group vs. UCMS + OIS100 group	0.003	
UMCS + vehicle group vs. UCMS + OIS500 group	< 0.001	
UCMS + OIS100 group vs. UCMS + OIS500 group	0.029	

Table S8. Paired Student's *t*-test and One-way analysis of variance (ANOVA) test of glucocorticoid receptor (GR) in frontal cortex and hippocampus

Group comparison	ANOVA followed by Tukey's post hoc test	
	P	**F(DF$_{between\ group}$, DF$_{residual}$)**
**Frontal cortex**		
non-stress group vs. UCMS + vehicle group	< 0.001	t(10) = 11.873
All UCMS-induced groups	< 0.001	F(3,20) = 27.307
UMCS + vehicle group vs. UCMS + IMP20 group	< 0.001	
UMCS + vehicle group vs. UCMS + OIS100 group	0.007	
UMCS + vehicle group vs. UCMS + OIS500 group	< 0.001	
UCMS + OIS100 group vs. UCMS + OIS500 group	0.001	
**Hippocampus**		
non-stress group vs. UCMS + vehicle group	< 0.001	t(10) = 10.531
All UCMS-induced groups	< 0.001	F(3,20) = 8.894
UMCS + vehicle group vs. UCMS + IMP20 group	0.005	
UMCS + vehicle group vs. UCMS + OIS100 group	0.363	
UMCS + vehicle group vs. UCMS + OIS500 group	< 0.001	
UCMS + OIS100 group vs. UCMS + OIS500 group	0.034	

Statistical Analysis of Effect of the OIS extract on UCMS-Induced Impaired Neurogenesis in Frontal Cortex and Hippocampus Using Quantitative real-time polymerase chain reaction (qPCR)

Table S9. Paired Student's *t*-test and One-way analysis of variance (ANOVA) test of brain-derived neurotrophic factor (BDNF) in frontal cortex and hippocampus

Group comparison	ANOVA followed by Tukey's post hoc test	
	P	**F(DF$_{between\ group}$, DF$_{residual}$)**
**Frontal cortex**		
non-stress group vs. UCMS + vehicle group	< 0.001	t(10) = 9.911
All UCMS-induced groups	< 0.001	F(3,20) = 27.347
UMCS + vehicle group vs. UCMS + IMP20 group	< 0.001	
UMCS + vehicle group vs. UCMS + OIS100 group	0.100	
UMCS + vehicle group vs. UCMS + OIS500 group	< 0.001	
UCMS + OIS100 group vs. UCMS + OIS500 group	< 0.001	
**Hippocampus**		
non-stress group vs. UCMS + vehicle group	< 0.001	t(10) = 9.214
All UCMS-induced groups	< 0.001	F(3,20) = 10.661
UMCS + vehicle group vs. UCMS + IMP20 group	0.007	
UMCS + vehicle group vs. UCMS + OIS100 group	0.750	
UMCS + vehicle group vs. UCMS + OIS500 group	< 0.001	
UCMS + OIS100 group vs. UCMS + OIS500 group	0.004	

Table S10. Paired Student's *t*-test and One-way analysis of variance (ANOVA) test of cyclic AMP-responsive element-binding protein (CREB) in frontal cortex and hippocampus

Group comparison	ANOVA followed by Tukey's post hoc test	
	P	**F(DF$_{between\ group}$, DF$_{residual}$)**
**Frontal cortex**		
non-stress group vs. UCMS + vehicle group	< 0.001	t(10) = 8.993
All UCMS-induced groups	< 0.001	F(3,20) = 13.720
UMCS + vehicle group vs. UCMS + IMP20 group	0.005	
UMCS + vehicle group vs. UCMS + OIS100 group	0.814	
UMCS + vehicle group vs. UCMS + OIS500 group	< 0.001	
UCMS + OIS100 group vs. UCMS + OIS500 group	< 0.001	
**Hippocampus**		
non-stress group vs. UCMS + vehicle group	< 0.001	t(10) = 4.649
All UCMS-induced groups	< 0.001	F(3,20) = 7.058
UMCS + vehicle group vs. UCMS + IMP20 group	0.031	
UMCS + vehicle group vs. UCMS + OIS100 group	0.911	
UMCS + vehicle group vs. UCMS + OIS500 group	0.004	
UCMS + OIS100 group vs. UCMS + OIS500 group	0.018	

Table S11. Validation result of the HPLC method for determination of baicalin, baicalein, chrysin, and oroxylin A

Parameter		Baicalin	Baicalein	Chrysin	Oroxylin A
LOQ	Concentration (μg/ml)	1.0	2.5	1	2.5
	S/N	9.83 ± 0.20	9.90 ± 0.19	9.84 ± 0.10	9.91 ± 0.13
Linearity	Range (μg/ml)	1 – 6	2.5 – 15	2.5 – 15	2.5 – 15
	Equation	y = 54.636x - 21.493	y = 51.014x - 54.144	y = 88.454x - 45.318	y = 72.566x - 76.601
	Coefficient determination (R^2)	0.9997	0.9996	0.9991	0.9997
Precision	Repeatability (within day) RSD	0.26 – 0.92%	0.1 – 1.59%	0.28 – 0.89%	0.08 – 0.80%
	Intermediate precision (between day) RSD	0.30 – 3.77%	0.20 – 1.32%	0.08 – 0.95%	0.14 – 1.43%
Accuracy (%recovery)	Low concentration	104.8 ± 1.43	101.66 ± 0.86	104.99 ± 0.71	101.40 ± 0.65
	Medium concentration	99.39 ± 0.12	99.95 ± 0.52	100.87 ± 0.094	100.66 ± 0.23
	High concentration	100.13 ± 0.15	99.92 ± 0.15	99.41 ± 0.085	99.48 ± 0.14

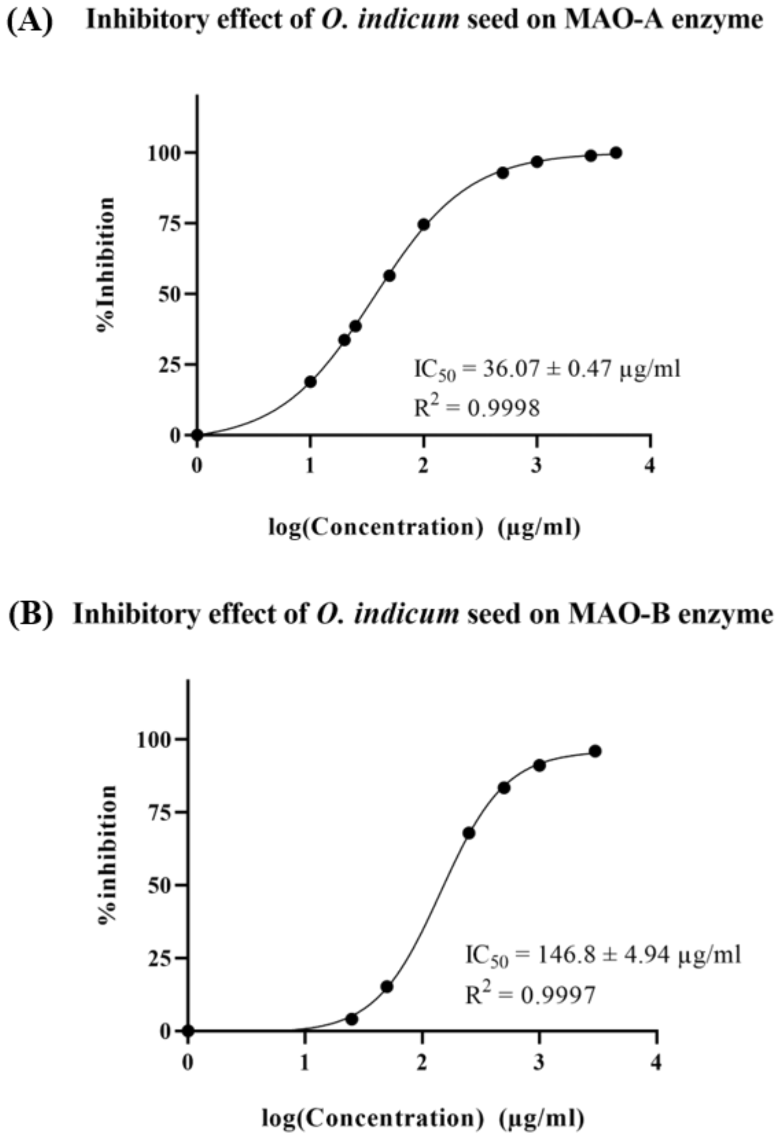

Figure S1. Inhibitory effect of *O. indicum* seed on MAO-A and MAO-B (panel A and B, respectively). The inhibition graph was plot between log(concentration) (X-axis) and %inhibition (Y-axis).

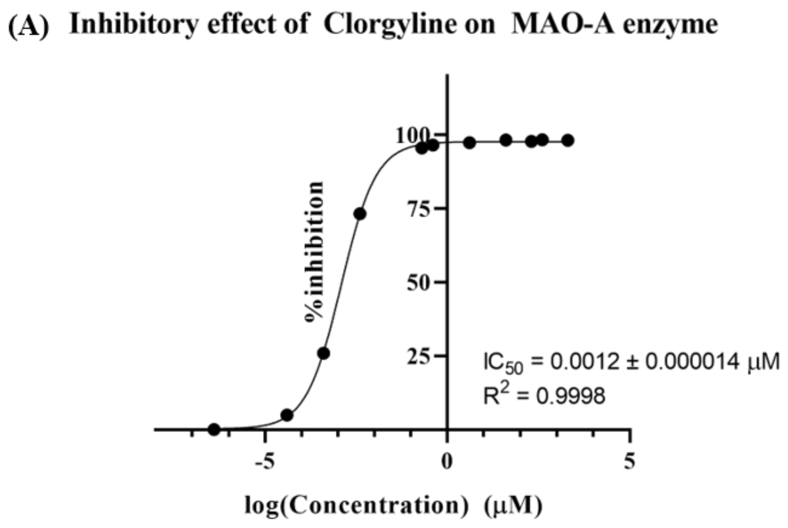

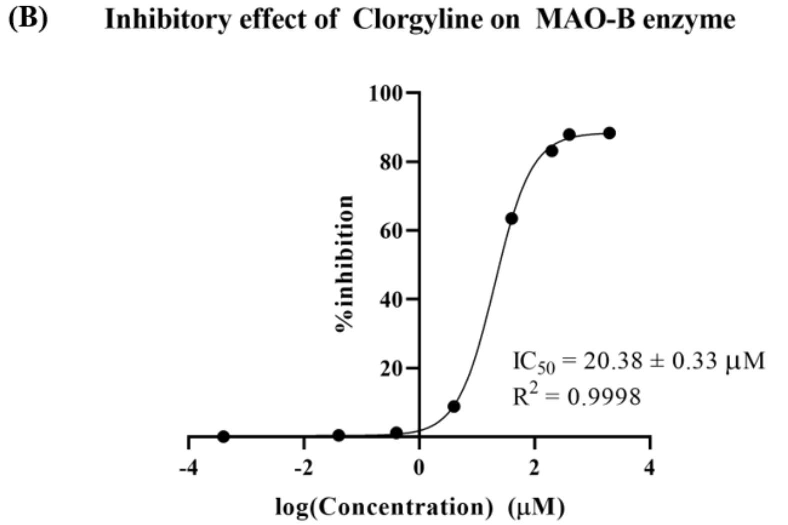

Figure S2. Inhibitory effect of Clorgyline on MAO-A and MAO-B (panel A and B, respectively). The inhibition graph was plot between log(concentration) (X-axis) and %inhibition (Y-axis)

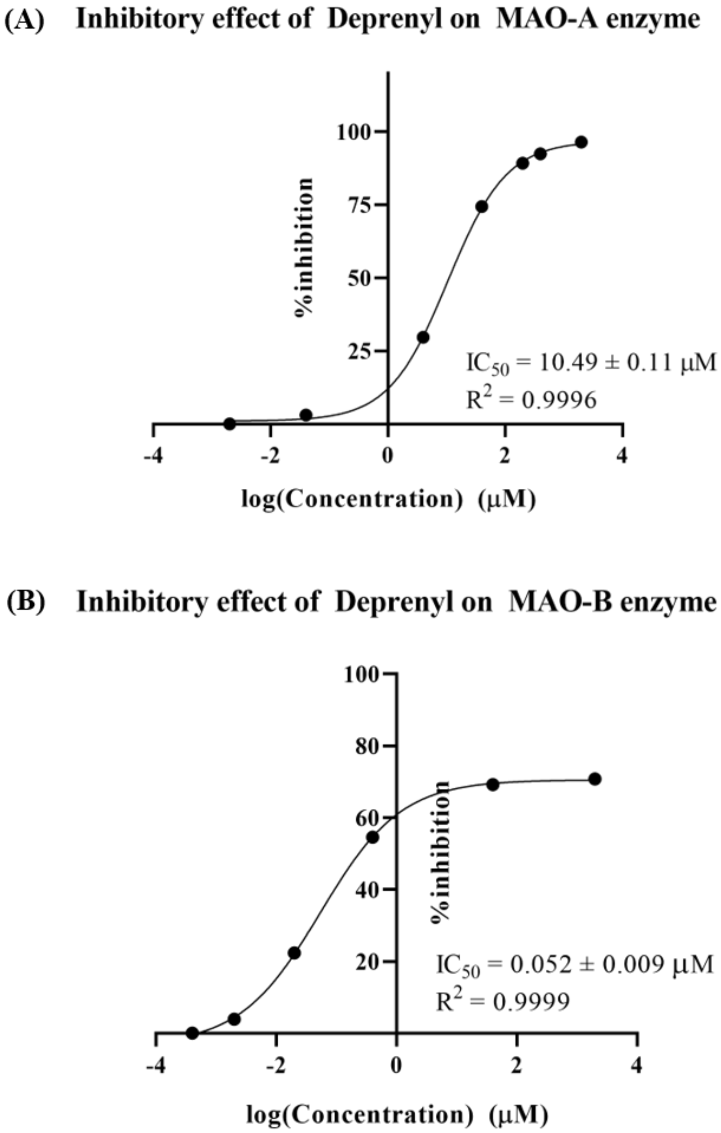

Figure S3. Inhibitory effect of Deprenyl on MAO-A and MAO-B (panel A and B, respectively). The inhibition graph was plot between log(concentration) (X-axis) and %inhibition (Y-axis)
